# Supplementary figures and images for: Combining environmental suitability and population abundances to evaluate the invasive potential of the tunicate Ciona intestinalis along the temperate South American coast
Source: PeerJ. 2015 Oct 27;3:e1357. doi: 10.7717/peerj.1357 (PMC4627925; doi:10.7717/peerj.1357)

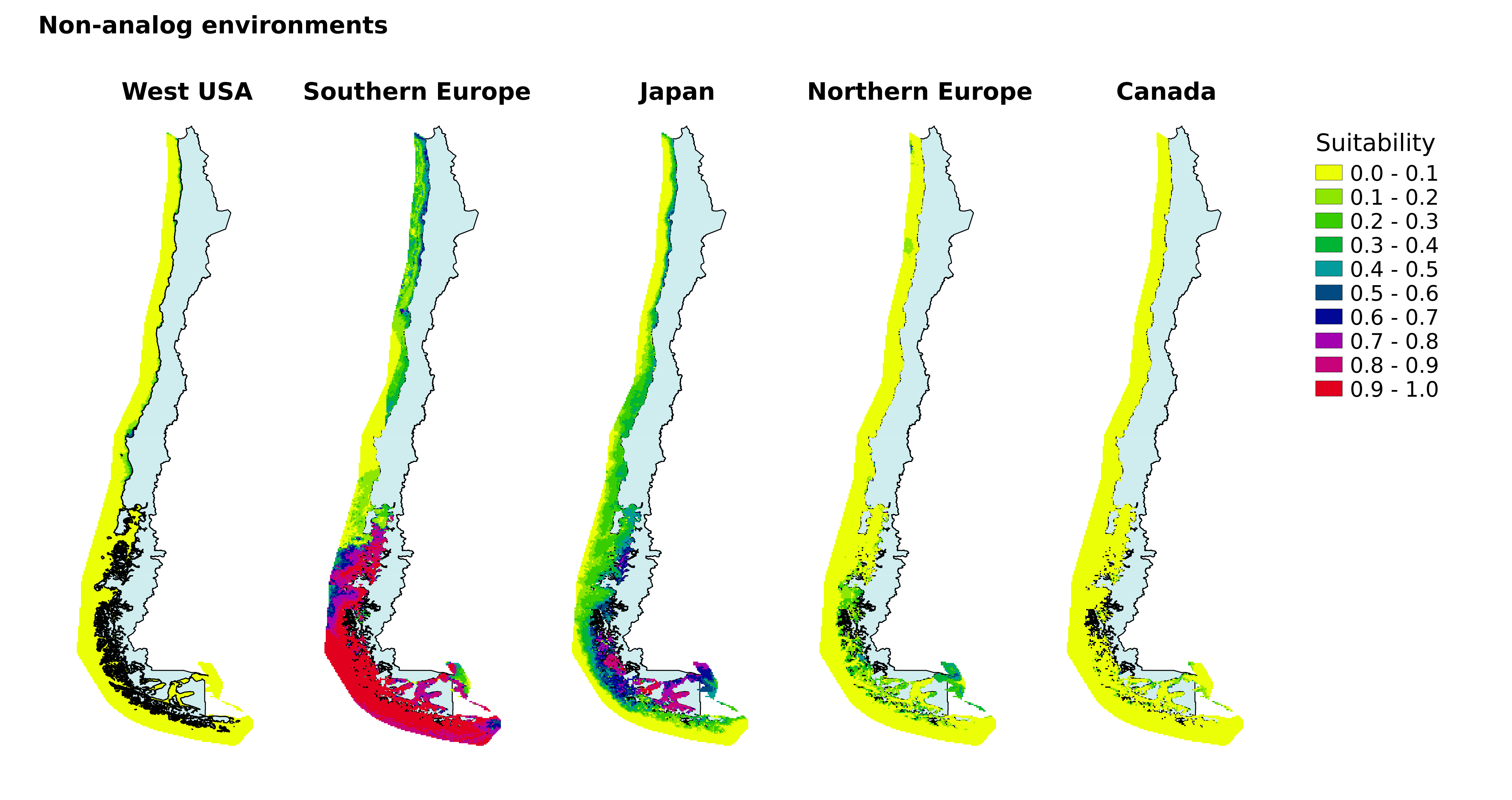

Supplement: Figure S1 — Projections of the potential distribution of C. intestinalis on Chilean coast using non analog environments for each of the five locations used in the analysis. [file peerj-03-1357-s001.png]

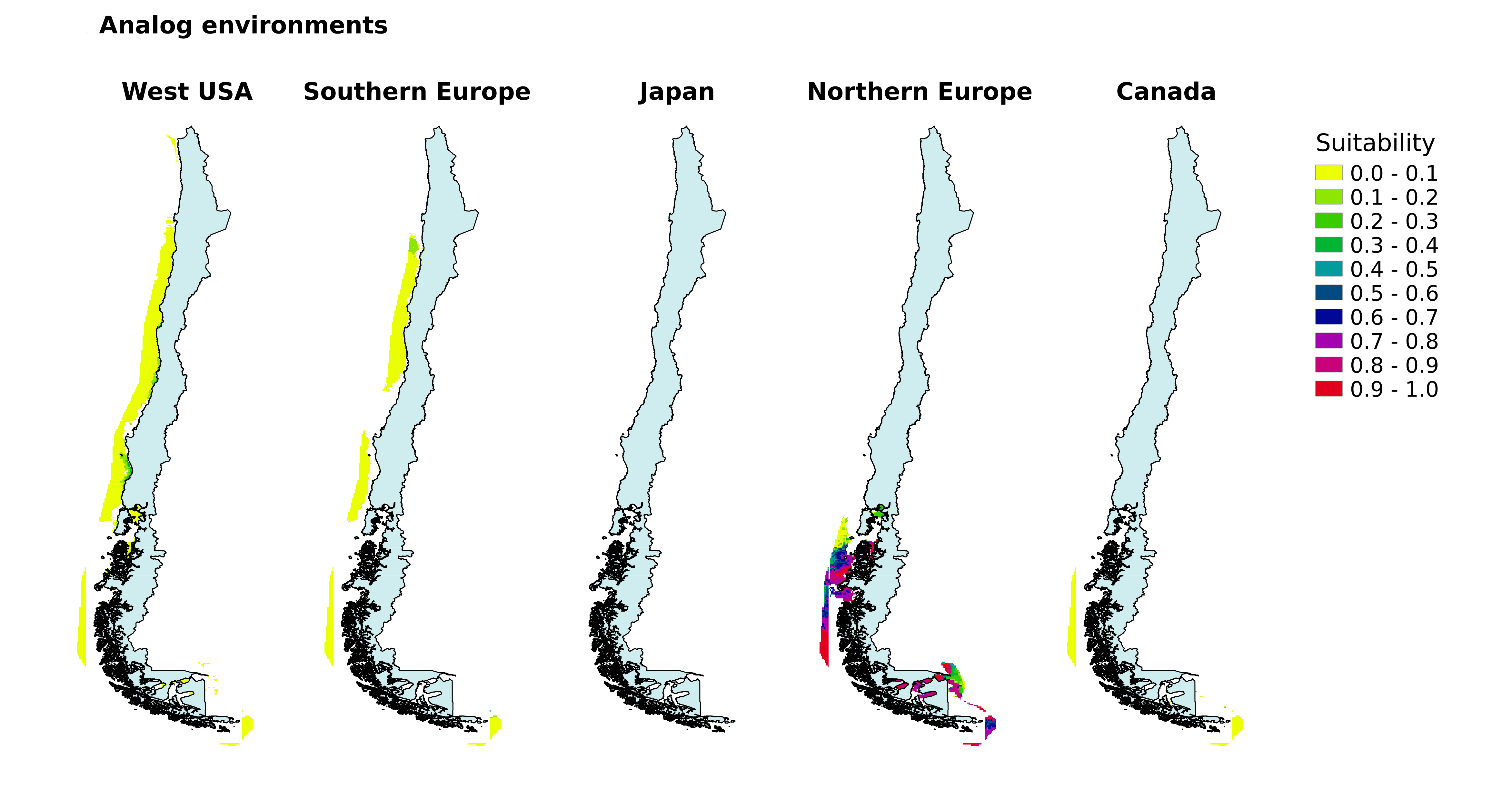

Supplement: Figure S2 — Projections of the potential distribution of C. intestinalis on Chilean coast using just analog environments for each of the five locations used in the analysis. [file peerj-03-1357-s002.png]
